# Comparison between pressure-recording analytical method (PRAM) and femoral arterial thermodilution method (FATD) cardiac output monitoring in an infant animal model of cardiac arrest

**DOI:** 10.1186/s40635-016-0087-0

**Published:** 2016-06-03

**Authors:** Javier Urbano, Jorge López, Rafael González, Sarah N. Fernández, María José Solana, Blanca Toledo, Ángel Carrillo, Jesús López-Herce

**Affiliations:** Pediatric Intensive Care Department, Hospital General Universitario Gregorio Marañón, Madrid, Spain; Instituto de investigación sanitaria del hospital Gregorio Marañón (IiSGM), Madrid, Spain; Universidad Complutense, Madrid, Spain; Research Network on Maternal and Child Health and Development II (REDSAMID II), Spanish Health Institute Carlos III, Madrid, Spain

**Keywords:** Pressure recording analytical method, Cardiac index, Children, Cardiac output, Cardiac arrest, Infant animal model

## Abstract

**Background:**

The pressure-recording analytical method is a new semi-invasive method for cardiac output measurement (PRAM). There are no studies comparing this technique with femoral artery thermodilution (FATD) in an infant animal model.

**Methods:**

A prospective study was performed using 25 immature Maryland pigs weighing 9.5 kg. Fifty-eight simultaneous measurements of cardiac index (CI) were made by FATD and PRAM at baseline and after return of spontaneous circulation. Differences, correlation, and concordance between both methods were analyzed. The ability of PRAM to track changes in CI was explored with a polar plot.

**Results:**

Mean CI measurements were 4.5 L/min/m^2^ (95 % CI, 4.2–4.8 L/min/m^2^; coefficient of variation, 27 %) by FATD and 4.0 L/min/m^2^ (95 % CI, 3.6–4.3 L/min/m^2^; coefficient for variation, 37 %) by PRAM (difference, 0.5 L/min/m^2^; 95 % CI for the difference, 0.1–1.0 L/min/m^2^; *p* = 0.003; *n* = 58). No correlation between both methods was observed (*r* = 0.170, *p* = 0.20). Limits of agreement were −2.9 to 4.0 L/min/m^2^ (−69.9 to 84.9 %). Percentage error was 80.6 %. Only 26.1 % of data points lied within an absolute deviation of ±30° from the polar axis.

**Conclusions:**

No correlation nor concordance between both methods was observed. Limits of agreement and percentage of error were high and clinically not acceptable. No concurrence between both methods in CI changes was observed. PRAM is not a useful method for measurement of the CI in this pediatric model of cardiac arrest.

## Background

Cardiac output (CO) is a parameter that evaluates global hemodynamic function. Its measurement is useful for diagnosis and for monitoring critically ill patients [1]. Early identification of important cardiovascular derangements can trigger promptly life support maneuvers which may avoid progression to cardiac arrest (CA) [2]. Asphyxia and other respiratory conditions are common causes of cardiac arrest in children [3]. Following an asphyxial event with sudden hypercapnia and hypoxia, several hemodynamic changes have been described [4]. Initial transient tachycardia with arterial hypertension was followed in the next minutes by progressive bradycardia and hypotension with low CO, pulmonary hypertension, tissue hypoxia, and lactic acidosis.

There are several invasive and noninvasive methods of measuring CO [5–15]. After recovery of spontaneous circulation (ROSC), it is of paramount importance to achieve an adequate hemodynamic status in order to protect the brain and organs from hypoperfusion [16]. Postresuscitation myocardial dysfunction (PRMD) is the most important aspect of postarrest shock and was first described by Laurent et al. [17]. Echocardiography is a useful tool to detect PRMD [5, 18]. However, it has some drawbacks, as for example, a trained operator is needed, and measurements are intermittent. In the settings where ecocardiography is not available, other cardiac output measurement devices may be useful to manage PRMD.

Less invasive methods for measuring cardiac output have been developed and validated in adults [5], and some of them have also been studied in children [7–13]. Conversely, nowadays, there is no continuous, reliable, and minimally invasive method for measuring cardiac output in children [5].

Femoral artery thermodilution (FATD) is a less invasive method that allows continuous CO measurement and calibration by transpulmonary thermodilution (TD), which has shown an acceptable reliability [7]. However, FATD needs a specific expensive catheter, and in hemodynamically unstable patients, it requires frequent recalibrations to obtain accuracy. For these reasons, it has not been in widespread use. Non invasive methods based in impedance, electrical velocimetry, ultrasound or ultrasound dilution have several limitations: intermittent measurements, operator dependency [5, 9, 10], lack of reliability [11–15], complexity and price [9]. Pulse contour analysis devices are based in the arterial pulse wave analysis and have the advantage of utilizing a preexisting arterial line, and most of them do not require external calibration. However, none of these methods has proven to be reliable in children [8].

Pressure recording analytical method (PRAM) is an invasive continuous (beat to beat) method based on the analysis of the morphology of both the pulsatile and continuous components of the arterial pressure waveform, at a higher sampling frequency, than the other pulse contour analysis technologies [19].

Previous studies have found a good correlation and concordance between PRAM and other methods for the measurement of cardiac output in animal models [20] and adults [21]. Nevertheless, some other studies have shown discordant results [22–25]. There are very few studies that have analyzed the utility of this technique in children and results are nonconcordant [14, 15, 26].

The objective of this study was to analyze the correlation, concordance, and the trending ability between pressure-recording analytical method and femoral artery thermodilution in a pediatric animal model of asphyxial CA.

## Methods

The experimental protocol was approved by the local Institutional Ethics Committee for Animal Research (permit number: 2013/0140). European and Spanish guidelines for ethical conduct in the care and use of experimental animals were applied throughout the study. The experiments were performed in the Department of Experimental Medicine and Surgery, Gregorio Marañon University Hospital, Madrid, Spain. All efforts were made to minimize suffering.

Twenty-four healthy 2- to 3-month-old Maryland pigs with a mean weight of 9.1 kg (95 % CI, 8.2–9.9 kg) participated in the study. Food was withdrawn the night before, although water was provided ad libitum. Initial anesthesia was performed with intramuscular administration of ketamine and atropine, followed by propofol, fentanyl, and atracurium for oral endotracheal intubation with a cuffed tube. Mechanical ventilation was provided by a Servo 900C (Siemens-Elema AB, Solna, Sweden) with 20 breaths per minute, tidal volume of 10 ml/kg, FiO2 of 35 %, and positive end-expiratory pressure (PEEP) of 3 cmH2O. Tidal volume was adjusted to achieve an end-tidal CO_2_ (EtCO_2_) from 33 to 35 mmHg and PaCO2 from 35 to 45 mmHg. Sedation and muscle relaxation (propofol 10 mg/kg/h, fentanyl 10 mcg/kg/h, and atracurium 2 mg/kg/h intravenous continuous infusion) was maintained throughout the procedure, in order to avoid the presence of spontaneous respiration. Monitoring included ECG, peripheral arterial hemoglobin oxygen saturation (Visconnet monitor, RGB Madrid, Spain), and the respiratory volumes and pressures, FiO2 and EtCO_2_, by means of a spirometer connected to the endotracheal tube and an S5 monitor (Datex Ohmeda, Madison, USA). A 4-F PiCCO catheter was inserted into the femoral artery to measure the blood pressure and CO by means of a femoral arterial thermodilution system (PiCCO, Pulsion Medical Systems, Munich, Germany). A Baxter Truwave PX-600 F transducer (Baxter Edwards, Irvien,CA, USA) was connected to the PRAM monitor (MostCare; Vytech Health, Padova, Italy). Both monitors were connected to the same femoral artery catheter using a three-way stopcock, as has been previously described [21]. A 5-F catheter was placed through the external jugular vein to measure the central venous pressure. The asphyxial cardiac arrest model has been described elsewhere [4]. Briefly, it mimics a hypoxic cardiac arrest, as occurs after an apnea, for example. When baseline data were collected, an intravenous bolus of atracurium was administered and CA was induced by disconnection from the respirator for at least 10 min. After this time, CA (defined as a mean arterial pressure less than 25 mmHg) was confirmed and then cardiopulmonary resuscitation (CPR) was started. CPR was performed as described elsewhere [4]. If ROSC was achieved, animals were observed without any intervention for 60 min, and sacrificed by the administration of a sedative overdose and the intravenous injection of potassium chloride on completion of the experiment.

### Comparison between FATD and PRAM

Cardiac output measurements were performed simultaneously by means of FATD and PRAM. The data recorded by the PRAM monitor (MostCare; Vytech Health, Padova, Italy) included cardiac index (CI), stroke volume index (SVI), systemic vascular resistance index (SVRI), and stroke volume variation (SVV). The data recorded by FATD (PiCCO, Pulsion Medical System, AG, Munich, Germany) included CI, SVRI, SVI, and SVV.

Intermittent CO and related parameters after thermodilution were obtained with FATD at baseline and 15, 30, and 60 min after ROSC (*ROSC15*’, *ROSC30*’, *ROSC60*’, respectively). Thermodilution was performed by the injection of 5 mL of ice-cold saline into a central vein over about 3 s. In each moment, two consecutive injections were completed in an interval of 30 s between each, approximately. If the series of CI measurements presented a discrepancy higher than 10 %, repeated injections were administered until satisfactory measurements were obtained. Beat to beat CO was obtained from PRAM by connecting the monitor to the femoral artery catheter using a three-way stopcock, as described above. Arterial pressure transducer system was zeroed at the midaxillary line. Two PRAM-CI measurements were registered simultaneously over the time when the FATD thermodilution was performed. The mean of the two measurements was considered for comparison. Measurements of the systolic arterial blood pressure with a difference higher than 10 % between FATD and PRAM were rejected. As recommended by the manufacturer, measurements with PRAM with a maximal pressure/time ratio (DP/dt) higher than 1.7 at baseline were also discarded because of a potential resonance of the waveform. Since the animal weight was lower than 20 kg, the “pediatric patient” mode was selected in the device.

### Statistical analysis

The sample size was adjusted to comparable comparison studies [14, 20–23]. No formal power analysis was calculated. The statistical analysis was performed by using the SPSS (version 16.0). An analysis was completed of the bias, correlation, and concordance between the measurements taken by FATD and PRAM, after checking the normal distribution of the sample with the Kolmogorov-Smirnov test. The coefficient of variation for both methods was calculated. The paired Student’s *t* test was selected to compare means and Spearman’s test to analyze correlations. Non-parametric Wilcoxon rank test and Pearson’s test were preferred when the variables did not adjust to the normal distribution. The Bland and Altman method [27] was used to compare the results of the different measurement techniques, calculating the mean (bias) ± standard deviation (as a measure of precision) of the differences between the values obtained with each method. The differences between each pair of values were plotted over the average for each pair. Limits of agreement (LoA) were calculated as the mean bias ± 1.96 times the standard deviation. The LoA expressed as a percentage were calculated regarding the values measured with the FATD method. As recommended previously [28], the percentage error was calculated as the LoA of the bias divided by the mean CI of both methods. A percentage error higher than 30 % was considered as clinically not acceptable. Differences were considered significant at a *p* value less than 0.05.

A polar plot was built to test the ability of PRAM to track changes in CI, as suggested elsewhere [29]. Briefly, polar plots have been recently proposed as a statistical method to compare CO monitor’s trending ability to a reference standard [29]. Polar plots include the direction and magnitude of changes. For this reason, they overcome the deficiencies of concordance analysis. A polar plot is a graph where vectors are represented. The vectors are defined by an angle and a size. The angle corresponds to the deviation from the perfect concurrence in tracking changes between the reference and the test method (that would be 0°). The size depends on the magnitude of the change and on the relationship between the change in CO from the two methods.

Changes in CI between consecutive pairs of thermodilution and PRAM measurements were analyzed. Data points representing changes <10 % of the mean CI (changes lower than 0.45 L/min/m^2^ in our study) were excluded for the analysis. Good concurrence was considered if 95 % or more of the points laid within an absolute deviation of ±30° from the polar axis, as previously recommended [14, 29].

In order to explore the differences between CI, SVI, and the heart rate (HR) detected by each monitor, a secondary analysis ad hoc was performed. The HR that the PRAM device was using at each moment in each subject to estimate CI was calculated by using the formula (CI*1000)*SVI^−1^. A comparison between calculated HR with the HR measured by the FATD monitor was performed by a paired Student’s *t* test.

## Results

**Fig. 1:**
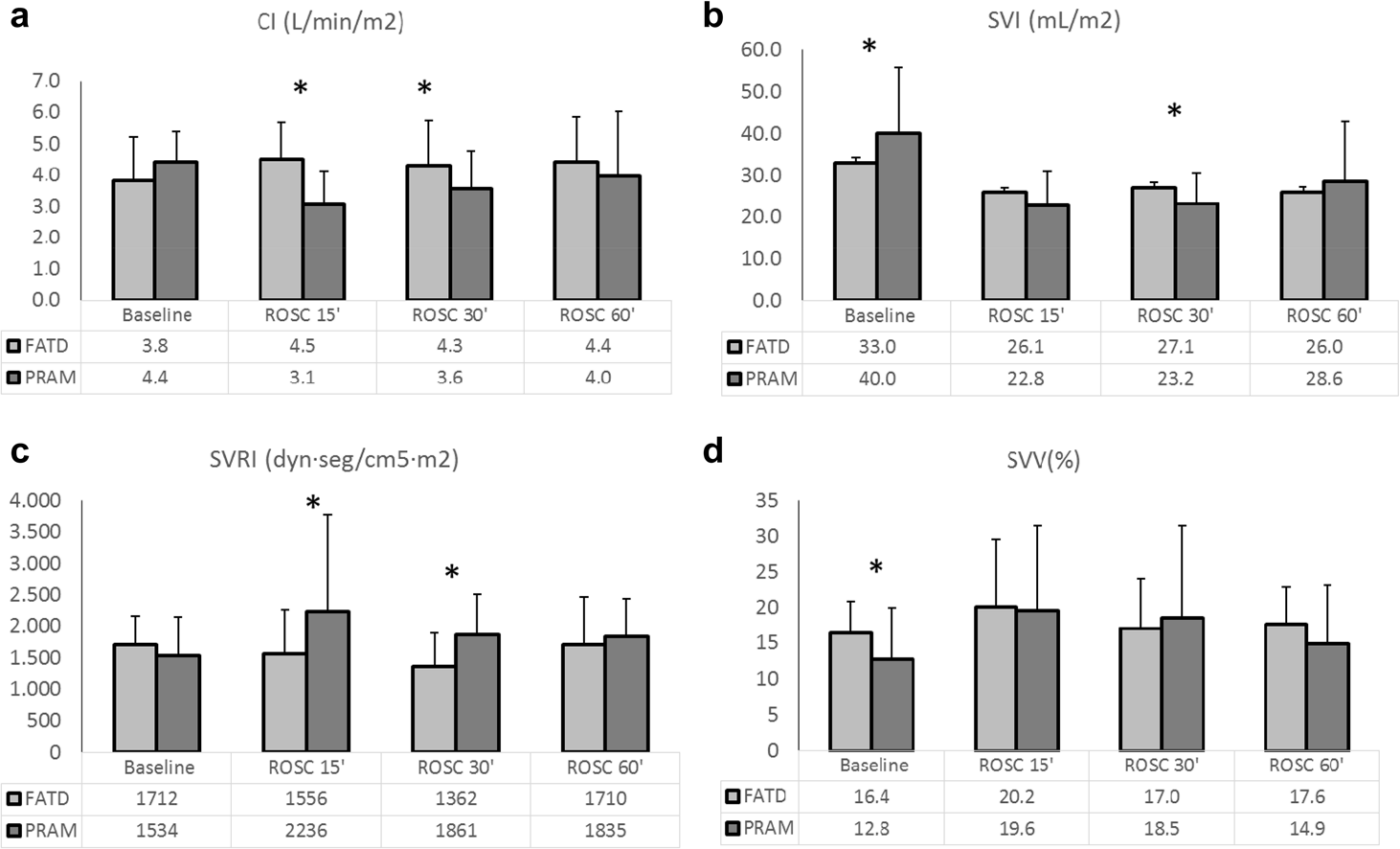
Evolution of cardiac index (**a**), stroke volume index (**b**), systemic vascular resistance index (**c**), and stroke volume variation (**d**) throughout the experiment, measured by femoral artery thermodilution (*FATD*), and the pressure-recording analytical method (PRAM). *p value <0.05

Fifty-eight pairs of measurements were obtained from 24 piglets and 12 piglets (50 %) achieved ROSC. Figure 1 represents the changes in CI, SVI, SVRI, and SVV measured at each of the study time points.

### Cardiac index

On combined analysis of all the paired measurements, the mean CI measurements were 4.5 L/min/m^2^ (95 % CI, 4.2–4.8 L/min/m^2^; coefficient of variation, 27 %) by FATD and 4.0 L/min/m^2^ (95 % CI, 3.6–4.3 L/min/m^2^; coefficient for variation, 37 %) by PRAM (difference, 0.5 L/min/m^2^; 95 % CI for the difference, 0.1–1.0 L/min/m^2^; *p* = 0.003; *n* = 58). No correlation between both methods was observed (*r* = 0.170, *p* = 0.20). When comparing the measurements at each of the moments (Fig. 1a), differences were observed at ROSC15’ (*p* = 0.004) and at ROSC30’ (*p* = 0.01) and trends towards statistically significant differences at ROSC60’ (*p* = 0.07).

Moderate correlations were observed at ROSC30’ (*r* = 0.618, *p* = 0.043).

**Fig. 2:**
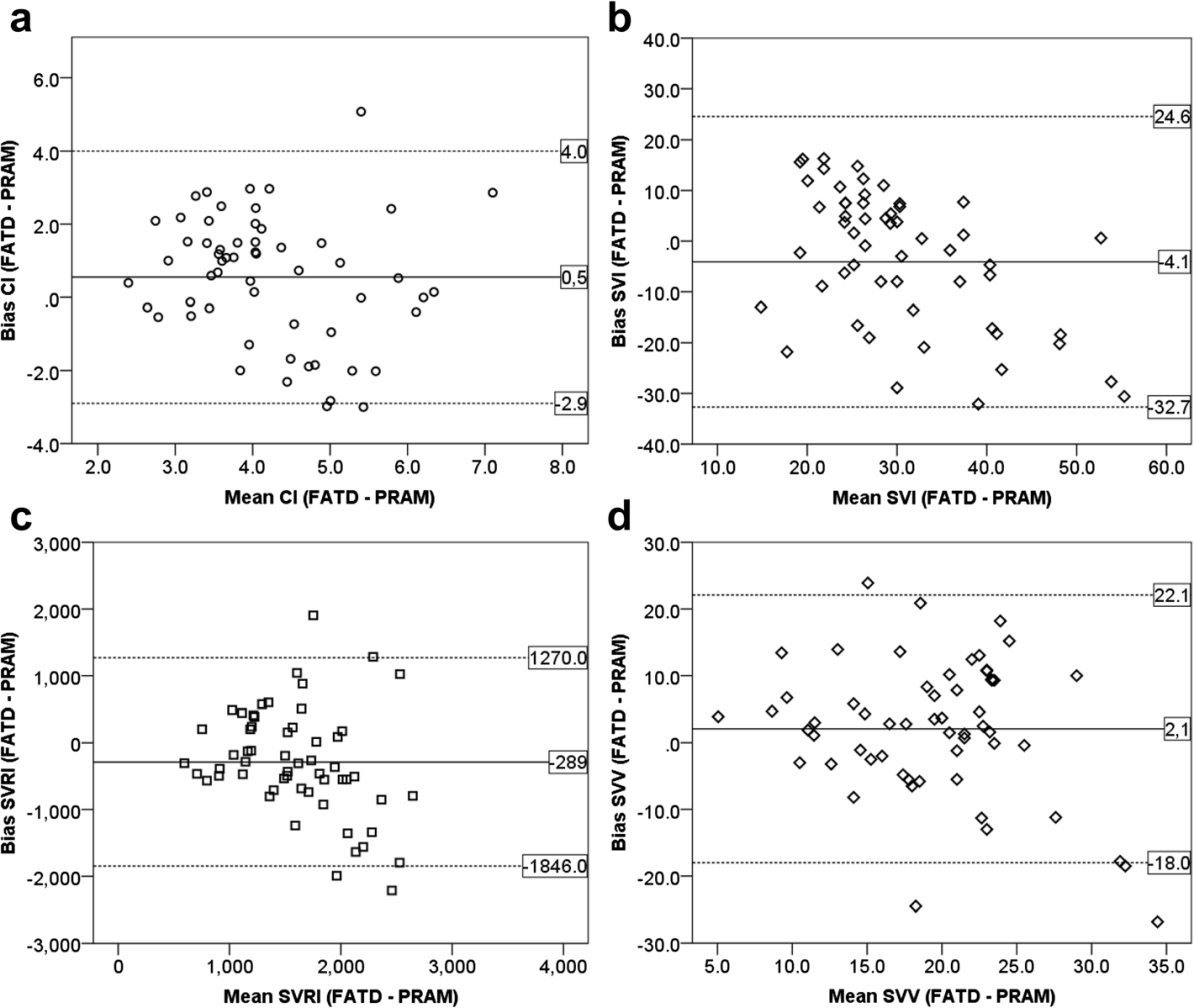
Bland and Altman plot differences between the cardiac index (**a**), stroke volume index (**b**), systemic vascular resistance index (**c**), and stroke volume variation (**d**) values obtained with femoral artery thermodilution (*FATD*) and the pressure-recording analytical method (*PRAM*)

The Bland Altman plot showed no agreement between the PRAM and FATD methods (Fig. 2a), with an overall percentage error of 80.6 %. The bias was 0.5 L/min/m^2^ (7.4 %), with a lower LoA of −2.9 and an upper LoA of 4.0 L/min/m^2^ (−69.9 to 84.9 %). The agreement at each of the four time points was similar (Table 1).

**Table 1:** Cardiac index measurements agreement analyzed by Bland-Altman’s approach at each study moment

Cardiac index FATD-PRAM (L/min/m^2^)	*N*	Bias	95 % CI	LoA high (%)	LoA low (%)	% error
Baseline	24	−0.4	−1.1 to 0.3	2.9 (67.4)	−3.7 (−97.7)	89.2
ROSC15’	11	1.8	1.2 to 2.4	3.9 (78.8)	−0.3 (−3.6)	48.3
ROSC30’	11	1.0	0.4 to 1.6	2.9 (63.5)	−0.9 (−19.6)	45.3
ROSC60’	12	0.9	−0.2 to 1.9	4.7 (82.7)	−2.9 (−58.9)	87.3

*FATD* femoral arterial thermodilution, *PRAM* pressure recording analytical method, *N* sample size, *Bias* mean of the differences between both methods, *95 % CI* 95 % confidence interval, *LoA (%)* limit of agreement expressed as absolute value and as the percentage of the reference method (FATD), *% error* percentage error, *ROSC* recovery of spontaneous circulation

### Stroke volume index

The overall mean SVI was 30.4 mL/m^2^ (95 % CI, 28.6–32.2 mL/m^2^) measured by FATD and 31.2 mL/m^2^ (95 % CI, 27.3–35.1 mL/m^2^) by PRAM (difference, −0.8 mL/m^2^; 95 % CI for the difference, −4.1–2.5; *p* = 0.62; *n* = 58). Moderate correlation was found (*r* = 0.552, *p* < 0.001). When comparing the measurements at each of the moments (Fig. 1b), differences were observed at baseline (*p* = 0.04) and at ROSC30’ (*p* = 0.03). Moderate correlation was observed only at baseline (*r* = 0.553, *p* = 0.005).

### Systemic vascular resistance index

The value for SVRI, on combined analysis of all measurements, was found to be lower using FATD: 1412 dyn*s*cm^−5^*m^−2^ (95 % CI, 1273–1561 dyn*s*cm^−5^*m^−2^) than when using PRAM: 1701 dyn*s*cm^−5^*m^−2^ (95 % CI, 1549–2025 dyn*s*cm^−5^*m^−2^), (difference, −288 dyn*s*cm^−5^*m^−2^; 95 % CI for the difference, −497–79 dyn*s*cm^−5^*m^−2^; *p* = 0.003; *n* = 58). No correlation was observed (*r* = 0.173, *p* = 0.19). There were significant differences on comparing the two methods at ROSC15’ (*p* = 0.02) and ROSC30’ (*p* = 0.04) (Fig. 1c). No correlations were observed.

### Stroke volume variation

Figure 1d shows the changes in the SVV. The value for SVV measured by FATD, using all measurements, was 17.6 % (95 % CI, 16.0–19.2 %), compared to 15.5 % (95 % CI, 13.0–18.1 %) by PRAM (difference, 2.1 %; 95 % CI for the difference, −0.6–4.7 %; *p* = 0.13; *n* = 58). Weak correlation was found (*r* = 0.269, *p* = 0.04). The differences between the two methods were statistically significant at baseline (*p* = 0.02). No correlations were detected specifically at any study point.

### Concordance of SVI, SVRI, and SVV

The Bland-Altman plots showed no agreement between FATD and PRAM for SVI, SVRI, and SVV (Fig. 2), with similar results at each of the four study points (Table 2); LoA were consistently higher than 30 % (SVI, 87 to −115 %; SVRI, 118 to −178 %; SVV, 149 to −140 %, respectively).

**Table 2:** Bland-Altman’s analysis of the agreement of hemodynamic measurements at each study moment

FATD-PRAM	*N*	Bias	95 % CI	LoA high (%)	LoA low (%)
SVI baseline (mL/m^2^)	24	−6.8	−12.4 to −1.2	20.8 (70.7)	−34.4 (−111)
SVI ROSC15’ (mL/m^2^)	11	4.7	−0.2 to 9.5	20.8 (80.9)	−11.3 (−52.1)
SVI ROSC30’ (mL/m^2^)	11	5.9	1.6 to 10.2	20.2 (69.9)	−8.5 (−30.9)
SVI ROSC60’ (mL/m^2^)	12	−0.2	−7.39 to 6.9	24.6 (92.3)	−25.0 (−97.7)
SVRI baseline (dyn*s*cm^−5^*m^−2^)	24	163	−132 to 459	1610 (86.0)	−1283 (−77.6)
SVRI ROSC15’ (dyn*s*cm^−5^*m^−2^)	11	−919	−1880 to 41	2266 (148)	−4104 (−315)
SVRI ROSC30’ (dyn*s*cm^−5^*m^−2^)	11	−565	−912 to −220	582 (62.7)	−1714 (−178)
SVRI ROSC60’ (dyn*s*cm^−5^*m^−2^)	12	−310	−633 to 13	808 (70.1)	−1428 (−137)
SVV baseline (%)	24	3.8	0.9 to 6.6	17.6 (104)	−10.1 (−62)
SVV ROSC15’ (%)	11	2.8	−5.8 to 11.4	31.3 (250)	−25.7 (−289)
SVV ROSC30’ (%)	11	−2.2	−10.1 to 5.7	24.0 (109)	−28.3 (−143)
SVV ROSC60’ (%)	12	2.0	−2.3 to 6.3	16.9 (93.5)	−12.9 (−68)

*FATD* femoral arterial thermodilution, *PRAM* pressure recording analytical method, *N* sample size, *Bias* mean of the differences, *95 % CI* 95 % confidence interval of the Bias, *LoA (%)* limit of agreement expressed as absolute value and as the percentage of the reference method (FATD), *SVI* stroke volume index, *ROSC* recovery of spontaneous circulation, *SVRI* systemic vascular resistance index, *SVV* stroke volume variation

### Tracking of changes

**Fig. 3:**
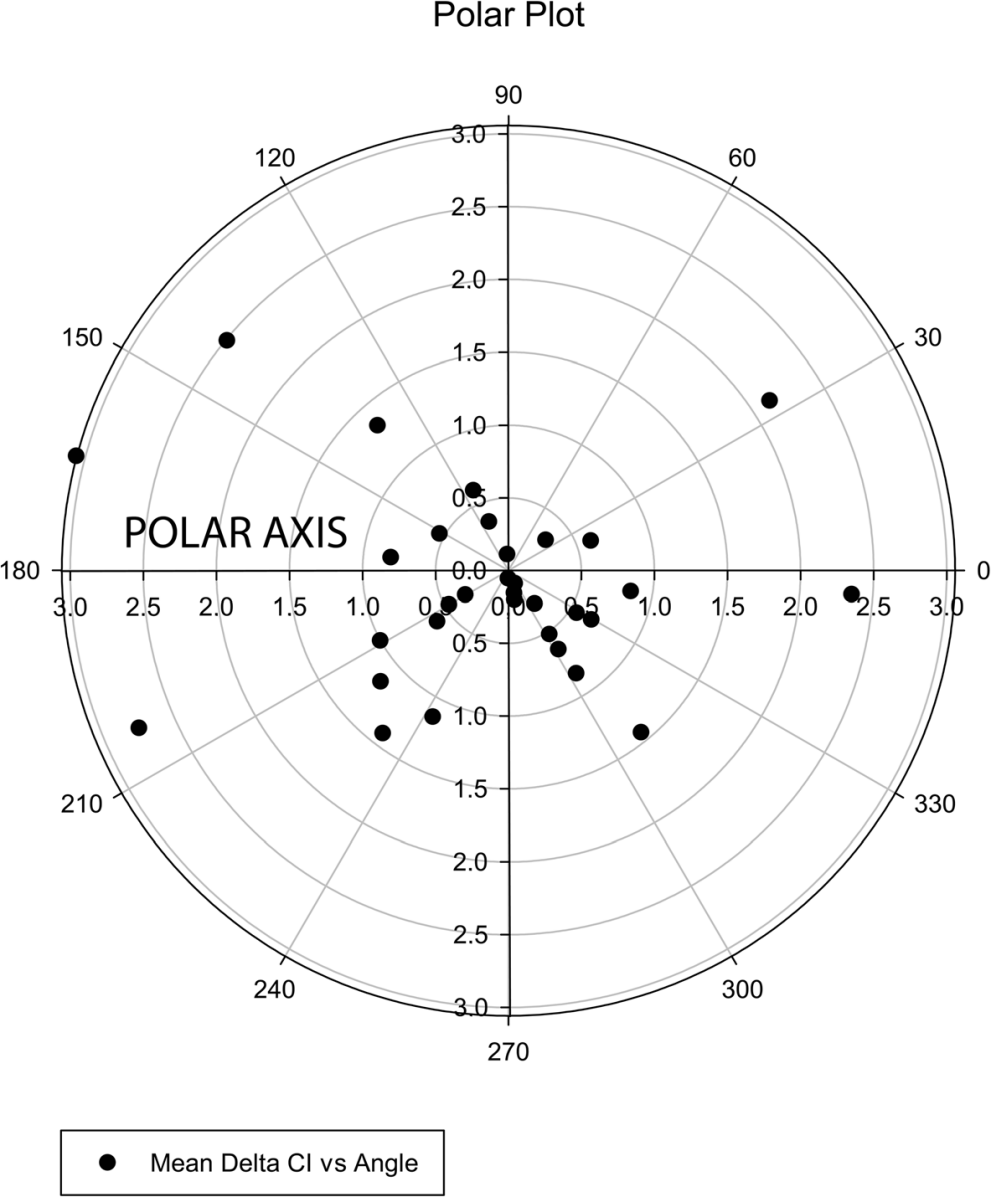
Polar plot to analyze the agreement between femoral artery thermodilution (FATD) and the pressure-recording analytical method (PRAM) for tracking changes in CI. Good agreement was measured by the proportion of data points (bold dots) falling within the polar limits of ±30° from the polar axis. Points lower than 0.45 L/min/m^2^ were disregarded for the analysis

The mean angular deviation from polar axis was −13.8°. Only 26.1 % of measurements fell within the polar limits of ±30° (Fig. 3).

### Differences between calculated and measured HR

The mean calculated HR used by the PRAM monitor to estimate CI was 138.5 bpm (95 % CI 126.7 to 150.3 bpm), and the HR detected by the FATD monitor was 153.1 bpm (95 % CI 141.1 to 165.1 bpm). The mean difference was −14.6 bpm (95 % CI for the difference, −28.2 to −1.0 bpm, *p* = 0.036). When comparing at each of the moments, differences were observed at ROSC15’ (*p* = 0.035) and at ROSC30’ (*p* = 0.045).

## Discussion

The evaluation and validation of cardiac output devices is much more complex in children than in adults. Most devices have been designed for adults and offer less accuracy and reliability when used in young children [8, 10–15].

The reference method to measure cardiac output in critically ill adult patients is the pulmonary artery thermodilution (PATD) using a pulmonary artery catheter (PAC). The use of the PAC has been reported previously in a hemorrhagic shock piglet model by our group [12]. However, severe complications occurred in a substantial number of animals, or was not possible to place the catheter in the correct position. On the other hand, the PAC is falling into disuse in the clinical practice in children, and we have considered that the FATD method was more suitable as the reference method. Despite its limitations, the most frequently used pulse contour technology in children is the PiCCO monitor (FATD method) [5–7], but it requires transpulmonary thermodilution to calibrate cardiac output, in addition to recalibration in cases of vasomotor tone and resistance changes. The PRAM, however, can measure absolute values of stroke volume independently of calibration using parameters that characterize the elastic properties of the arteries from an objective analysis of the pressure wave profile. Accordingly, PRAM does not require calibration and is both easy and fast to use.

While some studies in adults performed by the group who designed PRAM have shown a good correlation between this device and PATD, and with other several methods [19–21], others have reported discordant results. Paarmann et al. [23] found poor agreement between PRAM and PATD in 23 adults after cardiac surgery. Biais et al. [24] reported a high percentage of error on comparing PRAM and transthoracic echocardiography in 35 adult patients, and Maj et al. concluded that PRAM was not reliable in adult unstable patients with atrial fibrillation [25]. In children, Calamandrei et al. [26] compared CI measured by PRAM and Doppler echocardiography in 48 critically ill children and found a good level of correlation between both methods, with an acceptable percentage error of 21 %. In a recent study, Saxena et al. [14] compared 210 paired cardiac output measurements with PRAM and transpulmonary ultrasound dilution in 48 mechanically ventilated children. Although mean CI was similar with both methods, the LoA were wide (5.78 L/min/m^2^, with a percentage error of 143 %). Furthermore, the concordance between PRAM and transpulmonary ultrasound dilution was poor, with only 37 % of measurements falling within the predefined acceptable limits. The authors therefore concluded that PRAM was not recommendable for critically ill children. Finally, we have performed a clinical observational study in which a high percentage of CI measurements registered by PRAM in hemodynamically stable children were outside normal limits and might be influenced by age and weight of the patient [15].

Both pediatric studies and the study performed in adult patients that have shown poor reliability of PRAM [14, 15, 23] coincide in the absence of an exquisite selection of the arterial waveforms to be analyzed, as occurs in real daily practice, especially in emergency situations such as in our animal model.

To our knowledge, our study is the first to have investigated the validity of PRAM through its correlation and concordance with FATD in a pediatric animal model of cardiac arrest. We considered 20 % as the limit of agreement because that is the approximate variability of the reference method [28]. Our results indicate that there is no correlation or concordance between the CI measured by PRAM and that measured by FATD, as the mean of the differences was greater than 20 %, and percentage error was 104 %, similar to the study performed in children by Saxena et al. [14].

Interestingly, differences in CI were observed 15 min after ROSC despite that SVI was not significantly different between both methods. Considering that the heart rate (HR) of the subjects should be the same for both methods (because it was measured simultaneously), then the CI should not be different. There are two possible explanations for this fact. First, despite the mean difference between both methods was not statistically significant, the limits of agreement were wide. Positive and negative differences between both methods may be balanced, resulting in a small mean difference. And, second, the MostCare monitor may have missed some waveforms where dicrotic notch has not been detected to perform the calculations of the CI. Whereas the MostCare monitor measures the SVI in a waveform, and then calculates the CI with the rate of the waveforms detected, the PiCCO monitor measures the CI by thermodilution, regardless of the waveform. MostCare monitor depends on the detection of the dicrotic notch. If this point of the weaveform is not detected, the waveform is missed and does not count for the analysis. The two moments when the animals were typically more tachycardic were 15 and 60 min after ROSC, coinciding when the differences resulted statistically significant.

In this experiment, the PRAM monitor detected lower values of CI at 15 and 30 min after ROSC, whereas similar baseline values were measured by FATD. This fact may suggest that the changes over time measured by PRAM were more accurate than by FATD as typically after ROSC CI drops below baseline values [4, 17]. However, no statistically differences were observed between both methods at 60 min after ROSC.

Polar plots are recommended to evaluate the ability to track changes in CI, since Bland and Altman, and percentage of error analysis offer limited information. The polar plot demonstrated a poor ability of PRAM to track changes in CI, with a low percentage of data included within the acceptable limits. These results are similar to previously reported in children [14].

### Limitations

Our study has several possible limitations. First, the FATD is not the gold standard method to measure cardiac output. However, FATD in children has wide acceptance, as the PATD is a dangerous method in infants, because of the catheter placement. Second, we did not measure the dampening coefficient of the arterial line while using PRAM. Both under- and overdampening may affect the ability of PRAM to estimate CO [30]. However, from a clinician point of view during an emergency such as the period before and after a cardiac arrest, it is not realistic to spend time and efforts to test the accuracy of the arterial waveform. Nevertheless, arterial lines were flushed with 5 mL of saline if the arterial waveform was found to be dampened after a visual check, as we regularly do in daily clinical practice. And third, the sample size was small. This is an inherent limitation of experimental studies with large animals. Still, the width of the limits of agreement and the ability to track changes may have not changed despite a larger sample of animals was used.

## Conclusions

We conclude that PRAM is not a method comparable to femoral artery thermodilution for measurement of the CI in this pediatric model of cardiac arrest. This device should improve its algorithm for infants and children.

## Abbreviations

95 %, CI 95 % confidence interval; CA, cardiac arrest; CI, cardiac index; CO, cardiac output; CPR, cardiopulmonary resuscitation; ECG, electrocardiogram; EtCO_2_, end-tidal CO_2_; FATD, femoral artery thermodilution; FiO2, inspired fraction of oxygen; HR, heart rate; LoA, limit of agreement; PAC, pulmonary artery catheter; PATD, pulmonary artery thermodilution; PEEP, positive end-expiratory pressure; PRAM, pressure-recording analytical method; PRMD, postresuscitation myocardial dysfunction; ROSC, recovery of spontaneous circulation; SVI, stroke volume index; SVRI, systemic vascular resistance index; SVV, stroke volume variation; TD, thermodilution.
